# T-Cell Metabolic Reprogramming in Atherosclerosis

**DOI:** 10.3390/biomedicines12081844

**Published:** 2024-08-14

**Authors:** Shuye Chang, Zhaohui Wang, Tianhui An

**Affiliations:** Department of Geriatrics, Union Hospital, Tongji Medical College, Huazhong University of Science and Technology, Wuhan 430022, China; changshuye2024@163.com (S.C.); zhaohuiwang@hust.edu.cn (Z.W.)

**Keywords:** atherosclerosis, metabolic reprogramming, inflammations, T-cells

## Abstract

Atherosclerosis is a key pathological basis for cardiovascular diseases, significantly influenced by T-cell-mediated immune responses. T-cells differentiate into various subtypes, such as pro-inflammatory Th1/Th17 and anti-inflammatory Th2/Treg cells. The imbalance between these subtypes is critical for the progression of atherosclerosis (AS). Recent studies indicate that metabolic reprogramming within various microenvironments can shift T-cell differentiation towards pro-inflammatory or anti-inflammatory phenotypes, thus influencing AS progression. This review examines the roles of pro-inflammatory and anti-inflammatory T-cells in atherosclerosis, focusing on how their metabolic reprogramming regulates AS progression and the associated molecular mechanisms of mTOR and AMPK signaling pathways.

## 1. Introduction

Ischemic cardiovascular and cerebrovascular diseases, such as coronary heart disease, myocardial infarction, and stroke, are among the leading causes of death and disability in China [1]. Atherosclerosis (AS) serves as the primary pathological basis for these conditions [2]. It is well-established that AS is both a metabolic and a chronic inflammatory disease [3,4]. Helper T-cells (Th) and regulatory T-cells (Treg) play crucial roles in mediating the inflammatory response associated with AS [5]. Specifically, Th1 and Th17 cells exhibit pro-inflammatory effects, whereas Th2 and Treg cells demonstrate anti-inflammatory effects [6,7]. Research indicates that the balance between pro-inflammatory and anti-inflammatory responses, particularly between Th1/Th2 and Th17/Treg, dictates the progression or regression of AS.

In atherosclerosis (AS), T-cells demonstrate significant phenotypic plasticity. T-cell differentiation into pro-inflammatory (Th1, Th17) or anti-inflammatory (Th2, Treg) phenotypes depends on microenvironmental conditions, such as hyperlipidemia, hyperglycemia, hypoxia, oxidative stress, and the presence of apoptotic and necrotic cells [8,9]. Recent studies show [9] that hypoxia and high glucose levels activate T-cells’ glycolytic pathways, promoting differentiation into Th1 and Th17 phenotypes. Conversely, environments with ample oxygen and high lipid levels promote oxidative phosphorylation and fatty acid oxidation, leading to a preference for Th2 and Treg differentiation [10]. This change in crucial metabolic pathways, namely glycolysis, oxidative phosphorylation, and fatty acid oxidation, is known as “metabolic reprogramming” in immunometabolism [11,12]. This metabolic reprogramming alters the balance between Th1/Th2 and Th17/Treg differentiation in T-cells [13]. For example, transgenic mice overexpressing the glucose transporter 1 gene consume more glucose than their wild-type counterparts, leading to increased T-cell differentiation into Th1 and Th17 and reduced Treg differentiation [14]. Additionally, diets high in short-chain fatty acids promote more Treg differentiation and reduce inflammation [15]. Essentially, metabolic reprogramming is the key driver of the differentiation of plaque-resident T-cells (a specific subset of T-cells located within or near atherosclerotic plaques) into pro-inflammatory or anti-inflammatory phenotypes, shaping their fate and function and influencing AS progression or regression. This article discusses the roles of pro-inflammatory and anti-inflammatory T-cells in AS, emphasizing the crucial role of T-cell metabolic reprogramming in controlling AS development and its molecular mechanisms. It also reviews the burgeoning field of immunometabolism, exploring novel therapeutic advances for treating AS. We have synthesized and summarized the main findings of relevant studies in a comprehensive table (Table 1).

## 2. The Role of Pro-Inflammatory and Anti-Inflammatory T-Cells in Atherosclerosis

### 2.1. Pro-Inflammatory T-Cells Promote as Progression

Th1 cells, prevalent in the plaques, accelerate atherosclerosis progression by secreting pro-inflammatory cytokines [16], including interferon gamma (IFN-γ) and interleukin 2 (IL-2), (although IL-2 plays a role in the differentiation of Tregs, it can promote T-cell proliferation, activate NK cells, and increase their toxicity, exhibiting strong pro-inflammatory properties) [17,18,19]. Th1 cells and their pro-inflammatory secretions exacerbate plaque instability by inhibiting vascular smooth muscle cell proliferation and inducing macrophage differentiation into the pro-inflammatory M1 phenotype [20,21]. Clinical studies indicate a higher prevalence of Th1 cells in the plaques of patients post-stroke compared to those with asymptomatic atherosclerosis, implying that Th1 cells may facilitate plaque progression and potential rupture [16,22].

Th17 cells also play a pro-inflammatory and pro-AS role, mainly through the secretion of their characteristic cytokines IL-17 (interleukin-17, IL-17) and IL-6 (interleukin-6, IL-6) [5,6,8,9]. The number of Th17 cells is increased in patients with unstable angina or myocardial infarction compared to patients with stable angina and healthy subjects [19].

### 2.2. Anti-Inflammatory T-Cells Delay or Even Reverse Progression

Th2 cells, known for their anti-inflammatory properties, can delay or potentially reverse atherosclerosis progression, primarily via the secretion of cytokines like interleukin 4 (IL-4), interleukin 5 (IL-5), and interleukin 13 (IL-13) [23]. Research suggests that a lower presence of Th2 cells in individuals correlates with an increased likelihood of developing atherosclerotic formations [24].

Regulatory T-cells (Tregs) secrete anti-inflammatory cytokines like IL-10 and transforming growth factor-beta (TGF-β) [25], providing a comprehensive protective effect against atherosclerosis. Tregs regulate the differentiation of M1 to M2 macrophages, reduce monocyte infiltration, and enhance smooth muscle and collagen fiber proliferation, thereby stabilizing plaques and decelerating atherosclerosis progression through lipid reduction [26,27]. Preliminary and collaborative research confirms that patients with acute coronary syndrome exhibit reduced Treg cell counts, which are inversely associated with AS plaque stability [5]. Additionally, a large cohort study suggests that a lower blood ratio of Treg cells indicates a higher incidence of cardiovascular events.

Although some scholars recognize that Th2 cells may exhibit pro-inflammatory effects and Th17 cells may display anti-inflammatory effects [90,91,92], the majority of the literature supports the view that Th2 cells predominantly have anti-inflammatory properties, whereas Th17 cells are primarily pro-inflammatory.

Other studies have shown that certain biomarkers related to plaque stability, such as soluble suppressor of tumorigenicity (sST)-2, interleukin 33 (IL-33), and soluble fibrinogen-like protein 2 (sFgl2), can increase the differentiation of Treg cells within plaques, promote the shift from Th1 to Th2 cells, delay the progression of atherosclerosis, and exert a protective effect [93,94,95,96].

The roles of T helper cell subpopulations, like Th9, Th22, T follicular helper (Tfh)cells, and cytotoxic T-cells in atherosclerosis, are less studied compared to Th1, Th2, and Th17 cells [6]. Given the emerging nature of T-cell metabolic reprogramming research, we focused our manuscript on the more extensively studied T-cell subtypes—Th1, Th2, Th17, and Treg cells—and their roles in atherosclerosis.

**Figure 1:**
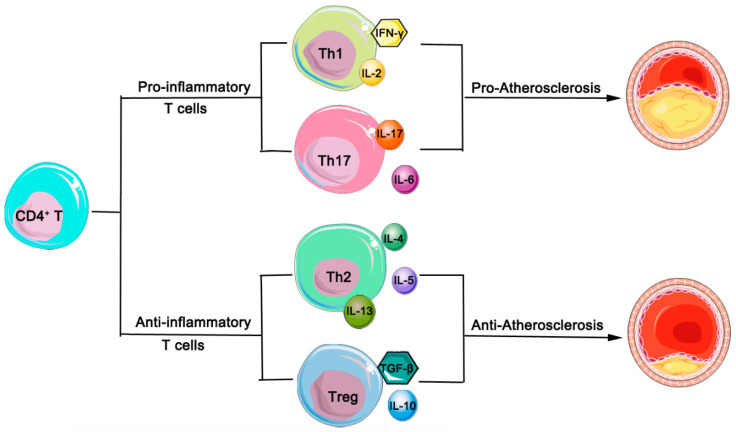
Simplified schematic of T-cell subsets and their roles in atherosclerosis. T-cells are broadly categorized into two major subsets: pro-inflammatory (Th1 and Th17 cells) and anti-inflammatory (Th2 and Treg cells). Th1 cells secrete pro-inflammatory cytokines, such as IFN-γ and IL-2, while Th17 cells contribute to inflammation by secreting IL-17 and IL-6. Conversely, Th2 cells produce anti-inflammatory cytokines, including IL-4, IL-5, and IL-13, and Treg cells secrete IL-10 and TGF-β, exerting anti-inflammatory effects. Pro-inflammatory T-cells are implicated in promoting the progression of atherosclerosis, whereas anti-inflammatory T-cells play a role in mitigating this progression. Abbreviations: Th cells, T-helper-cells; Treg, regulatory T-cells; IFN-γ, interferon-γ; IL, interleukin; TGF-β, transforming growth factor beta.

To enhance clarity, we have created a figure that provides a detailed depiction of the relationship between pro-inflammatory T-cells, anti-inflammatory T-cells, and the progression of atherosclerosis (Figure 1).

## 3. T-Cell Metabolic Reprogramming in Regulating Atherosclerosis Progression

### 3.1. Main Metabolic Pathways of Immune Cells

Like other cells, the metabolism of immune cells is essential for their survival and function. Common metabolic pathways in immune cells encompass glycolysis, oxidative phosphorylation (OXPHOS), the tricarboxylic acid (TCA) cycle, the pentose phosphate pathway (PPP), fatty acid oxidation (FAO), fatty acid synthesis (FAS), and amino acid metabolism. Glycolysis and oxidative phosphorylation (OXPHOS) are the primary mechanisms through which immune cells generate ATP [97,98]. In oxygen-rich conditions, cells primarily metabolize glucose via oxidative phosphorylation (OXPHOS), while glycolysis is favored under hypoxic conditions [99]. Interestingly, activated T-cells intensify aerobic glycolysis, even in oxygen-rich environments [100]; this process, though less energy-efficient, is advantageous for synthesizing critical biochemical intermediates [101]. A key glycolytic product, glucose-6-phosphate, is converted into fructose-6-phosphate, which either contributes to glycogen synthesis or enters the pentose phosphate pathway (PPP) [102]. The pentose phosphate pathway (PPP) supplies raw materials for nucleic acid synthesis and produces nicotinamide adenine dinucleotide phosphate (NADPH), essential for driving metabolic reactions [103].

### 3.2. Metabolic Reprogramming of Pro-Inflammatory and Anti-Inflammatory T-Cells in Atherosclerosis Progression

**Figure 2:**
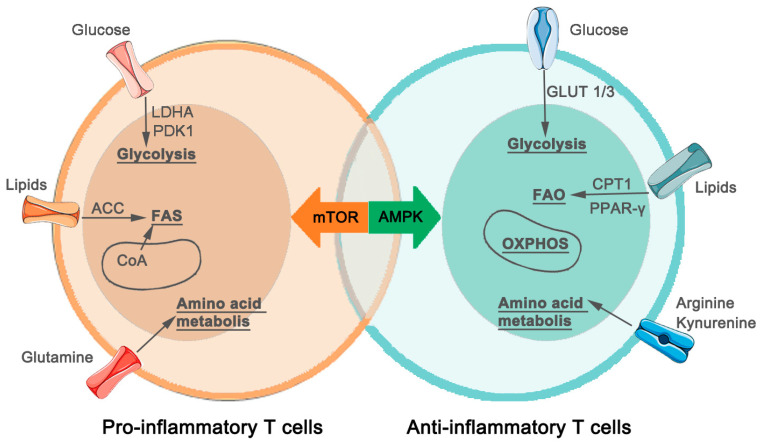
Metabolic characteristics of CD4 T-Cells in atherosclerosis. T-cells undergo significant metabolic reprogramming in the context of atherosclerosis. Pro-inflammatory T-cells, such as type 1 helper T-cells (Th1) and type 17 helper T-cells (Th17), exhibit a metabolic preference for glycolysis, fatty acid synthesis (FAS), and glutamine amino acid metabolism. This metabolic reprogramming provides these T-cells with energy and biosynthetic precursors necessary for their effector functions. The key enzymes involved in glycolysis and fatty acid synthesis are shown in the diagram. In contrast, regulatory T-cells (Tregs) and type 2 helper T-cells (Th2), which possess anti-inflammatory and atheroprotective properties, display metabolic characteristics marked by oxidative phosphorylation (OXPHOS), fatty acid oxidation (FAO), and glycolysis, with increased metabolism of arginine and kynurenine. These metabolic features support the suppressive functions of Tregs and Th2 cells, aiding in the maintenance of immune tolerance and the reduction of atherosclerotic inflammation. The key molecules involved in glycolysis and fatty acid oxidation are illustrated in the diagram. Abbreviations: LDHA, Lactate Dehydrogenase A; PDK1, Pyruvate Dehydrogenase Kinase 1; ACC, Acetyl-CoA Carboxylase; GLUT 1/3, glucose transporter 1/3; CPT1, Carnitine Palmitoyl transferase I; PPAR-γ, peroxisome proliferator-activated receptor γ.

We have depicted the characteristics of metabolic reprogramming in CD4 T-cells in the model diagram (Figure 2).

#### 3.2.1. Pro-Inflammatory T-Cells

##### Glucose Metabolic Reprogramming

The glucose metabolic reprogramming of pro-inflammatory T-cells plays a critical role in the pathophysiology of atherosclerosis (AS), attracting significant research attention. In atherosclerosis, Th1 and Th17 cells increase glucose uptake and utilization to satisfy the elevated energy demands of their activated state [28]. Research shows that [29] inflammatory stimuli significantly boost the glycolysis rate in these cells. Lactate dehydrogenase A (LDHA), essential for aerobic glycolysis, plays a pivotal role in sustaining this metabolic pathway [30]. LDHA activity directly impacts glucose consumption and lactate production [31]. Th1 and Th17 cells deficient in LDHA show decreased glucose consumption and lactate production, alongside an increase in oxidative phosphorylation (OXPHOS) [32,33]. This shift likely results from increased pyruvate input into the mitochondrial tricarboxylic acid (TCA) cycle, shifting their metabolic focus from rapid glycolysis to sustained energy production via OXPHOS, impacting their pro-inflammatory capabilities and, thus, influencing AS progression.

##### Lipid Metabolic Reprogramming

In the context of atherosclerosis (AS), the reprogramming of lipid metabolism in pro-inflammatory T-cells is pivotal to both the initiation and progression of the disease. Specifically, Th1 and Th17 cell subsets augment fatty acid synthesis pathways, supplying essential lipids for cellular biosynthesis and energy metabolism, and playing a significant role in regulating cell differentiation and function [34,35].

Under hypercholesterolemic conditions, the metabolic reprogramming of immune cells can result in an enhanced differentiation of pro-inflammatory Th1 and Th17 cells, while concurrently inhibiting the differentiation of regulatory T-cells (Tregs) [36]. This shift is particularly critical in the pathogenesis of atherosclerosis, as an increase in Th1 cells intensifies inflammatory responses, whereas a reduction in Tregs weakens immunosuppressive capabilities.

Furthermore, excessive cholesterol intake has been shown to promote Th17 cell differentiation [37,38], while excessive intake of long-chain fatty acids may enhance the differentiation and proliferation of pathogenic Th1 and Th17 cells [38,39,40]. Statins, by reducing blood cholesterol levels, have demonstrated the capacity to limit Th17 cell differentiation, thus playing a crucial role in immune regulation [41].

Acetyl-CoA Carboxylase (ACC) is a critical regulatory enzyme in fatty acid synthesis, catalyzing the conversion of Acetyl-CoA to Malonyl-CoA, thereby initiating the fatty acid synthesis process [42]. During T-cell activation and proliferation, cholesterol and its derivatives serve as vital components for cell membrane construction and also participate in the regulation of intracellular signaling and gene expression through interactions with specific transcription factors [43].

Notably, the activity of ACC1 is essential for Th17 cell differentiation. T-cells deficient in ACC1 are predisposed to differentiate into regulatory T-cells (Tregs) rather than Th17 cells [44]. Moreover, studies in mice have corroborated that inhibition of ACC1 activity results in a decreased production of Th17 cells, thereby modulating the balance of immune responses [44].

These findings underscore the pivotal role of ACC1 in T-cell differentiation and elucidate the potential implications of the fatty acid synthesis pathway in immune regulation.

##### Amino Acid Metabolic Reprogramming 

The reprogramming of amino acid metabolism plays a crucial role in the function of pro-inflammatory T-cells [45]. Subsets, such as Th1 and Th17 cells, rely on amino acid metabolism to provide essential precursors for the synthesis of cytokines and other biomolecules [46]. Under specific physiological or pathological conditions, alterations in the amino acid metabolism patterns of T-cells can significantly impact the progression of atherosclerosis (AS) [47].

In vitro studies have observed that glutamine deprivation leads to significant inhibition of activated T-cell growth, proliferation, and cytokine production [48]. Furthermore, in autoimmune mouse models, reduced expression of amino acid transporters has been associated with decreased differentiation of pro-inflammatory Th1 and Th17 cells [49]. This reduction in differentiation may lead to a diminished inflammatory response, providing strong evidence for the role of amino acid metabolism in regulating immune responses. Research indicates that insufficient leucine intake primarily restricts Th17 cell differentiation, with minimal impact on Th1 cell differentiation [50].

#### 3.2.2. Anti-Inflammatory T-Cells

##### Glucose Metabolic Reprogramming

The differentiation of T-cells is highly dependent on glycolysis, with Th2 cells expressing the highest levels of glucose transporter 1 (GLUT1) and exhibiting the most robust glycolytic activity [51,52]. Although Treg cells primarily obtain energy through mitochondrial lipid oxidation, glucose metabolism is also essential for their differentiation, function, and migration [53,54]. In vitro studies have shown that during the differentiation of naive T-cells into Treg cells, the expression of glucose transporters GLUT1 and GLUT3 is upregulated, leading to increased glucose uptake and consumption [55,56].

This metabolic reprogramming enhances glycolysis and promotes an increase in Treg cell numbers. However, it has also been observed to decrease the expression of Foxp3, a key transcription factor for the immunosuppressive function of Treg cells. Therefore, increased glucose metabolism may partially inhibit the immunosuppressive function of Treg cells [55,57].

The dual-edged effect of glucose metabolic reprogramming suggests that the metabolic state of Treg cells has a complex and finely tuned impact on their function. In developing therapeutic strategies for atherosclerosis (AS), it is crucial to carefully balance Treg cell proliferation, migration, and immunosuppressive function to achieve optimal therapeutic outcomes.

##### Lipid Metabolic Reprogramming 

Lipid metabolic reprogramming is crucial for the activation, proliferation, and growth of anti-inflammatory T-cells. The metabolic flexibility of Th2 cells and regulatory T-cells (Tregs) significantly influences their immunoregulatory functions and control of inflammatory responses. During Th2 cell differentiation, specific expression of peroxisome proliferator-activated receptor gamma (PPARγ) plays a pivotal role in regulating fatty acid metabolism [58]. Research indicates that statins [59], by reducing cholesterol levels, can enhance Th2 cell differentiation, potentially through impacts on membrane cholesterol content and signaling pathways. Additionally, administering squalene to mice increases membrane cholesterol enrichment and promotes Th2 differentiation by co-localizing signaling molecules within lipid rafts—namely interleukin-1 receptor antagonist (IL1Rα), IL2Rα, and IL4Rβ12—thereby enhancing the phosphorylation of the signal transducer and activator of transcription 4 and transcription activator 5 [43].

In Treg cells, mitochondrial fatty acid oxidation (FAO) serves as the primary energy source in their resting state, facilitated by oxidative phosphorylation (OXPHOS) [34,35,60]. This energy pathway not only sustains their regulatory functions but also enables rapid response during immune activation. Reprogramming of fatty acid synthesis and FAO during Treg cell differentiation is essential for preserving their immunosuppressive capabilities. Treg cells engage in de novo lipid synthesis via heightened expression of sterol regulatory element-binding protein (SREBP) [61] and fatty acid synthase (FASN), crucial for sustaining their immunosuppressive function.

Some research indicates that CPT1a, a key isoform of carnitine palmitoyltransferase I in lymphocytes, could influence Treg cell counts or Foxp3 expression levels, suggesting that Treg cell differentiation might rely on CPT1a-mediated fatty acid oxidation (FAO) [62]. This suggests that the metabolic flexibility of Treg cells is likely more complex than previously understood and could involve various metabolic pathways.

##### Amino Acid Metabolic Reprogramming 

Amino acid metabolism is crucial for the differentiation and functionality of anti-inflammatory T-cells. Th2 cell differentiation and function are linked to the tryptophan metabolism pathway [63]. Tryptophan metabolites, notably kynurenine, influence Th2 cell activity and immune regulation via the aryl hydrocarbon receptor (AHR) signaling pathway [64]. Amino acid metabolic reprogramming in Th2 cells includes polyamine synthesis, particularly spermine and spermidine, which are vital for proliferation and function of the cells [104,105]. These polyamines regulate gene expression through effects on intracellular signaling and epigenetic modulation, thus influencing Th2 cell differentiation and effector functions.

Amino acid metabolism is essential for the differentiation and function of regulatory T-cells (Tregs). L-arginine and its metabolite putrescine significantly promote the differentiation of Treg cells, both in vitro and in vivo, enhancing their ability to secrete the anti-inflammatory cytokine IL-10 [106]. This process plays a pivotal role in maintaining immune homeostasis and suppressing inflammatory responses, particularly in chronic inflammatory diseases like atherosclerosis (AS).

Additionally, kynurenine, a byproduct of tryptophan catabolism, contributes to the differentiation of Treg cells. The accumulation of kynurenine promotes the differentiation of T-cells into Treg cells, which helps alleviate inflammatory responses in atherosclerosis (AS) and provides a protective effect [64]. This underscores the intricate role of amino acid metabolism in shaping the immune landscape, highlighting potential therapeutic targets to modulate immune responses in inflammatory diseases.

## 4. Key Molecular Mechanisms of Pro-Inflammatory and Anti-Inflammatory T-Cell Metabolic Reprogramming

The metabolic processes in cells involve various signaling pathways, with mTOR and AMPK being critical molecules in regulating the role of T-cell metabolic reprogramming in the progression of atherosclerosis (AS).

### 4.1. mTOR

mTOR (mammalian target of rapamycin) serves as a central regulatory factor in T-cell metabolic reprogramming, operating through two complexes, mTORC1 and mTORC2 [65]. During T-cell differentiation and maturation, mTORC1 activation enhances glycolytic metabolism, cell growth, and the cell cycle [66]. Following T-cell receptor (TCR) stimulation, mTORC1 collaborates with the transcription factor c-Myc to boost the expression of glucose transporters and CD98, thus facilitating the uptake of glucose and amino acids [67].

During the initial phases of T-cell activation, mitochondrial biogenesis is regulated by Pgc1α (peroxisome proliferator-activated receptor gamma coactivator 1-alpha). Concurrently, there is an increased cellular demand for serine, which is metabolized via the mitochondrial serine hydroxymethyl transferase pathway to support glutathione synthesis [68,69]. Glutathione is crucial in limiting the buildup of reactive oxygen species (ROS) [70,71,72], thus facilitating T-cell activation. CD4+ T-cells differentiate into subgroups with unique functions and metabolic requirements, with the mTOR signaling pathway playing a critical role in regulating their metabolic reprogramming. Specifically, pro-inflammatory T-cell subgroups such as Th1 and Th17 depend on selective regulation by mTOR complex 1 (mTORC1) [73,74], whereas the Th2 subgroup is regulated by mTOR complex 2 (mTORC2) [75]. Inhibiting mTOR can skew differentiation toward Treg cells expressing FoxP3, underscoring the critical role of mTOR in shaping the fate and function of various T-cell subtypes within the immune system [76].

### 4.2. AMPK

The AMP-activated protein kinase (AMPK) complex, a serine/threonine kinase, serves as an energy metabolism sensor in the body [77,78]. AMPK activation is triggered by T-cell receptor (TCR) signaling through two distinct pathways [79,80]. The first pathway depends on liver kinase B1 (LKB1) and activates AMPK when the intracellular AMP/ATP ratio increases. This activation promotes catabolic metabolism and inhibits anabolic processes, thereby replenishing ATP production [81]. The second pathway involves activation through calcium influx, which triggers calmodulin-dependent protein phosphatase and subsequent activation of calcium/calmodulin-dependent kinases [82].

AMPK activation is mediated by various upstream protein kinases. During early T-cell activation, an increase in intracellular calcium activates AMPK, which then limits mTOR signaling and curbs the early involvement of glycolytic synthetic metabolism by inhibiting downstream mediators [83]. T-cells deficient in AMPK demonstrate a reliance on glycolysis and fail to re-engage mitochondria during glucose consumption, underscoring this concept [84]. AMPK activation inhibits both glycolysis [85] and fatty acid synthesis [86]. Conversely, AMPK enhances fatty acid oxidation by upregulating the expression and phosphorylation of CPT1a [87]. During T-cell activation, various upstream regulators modulate AMPK activity, and the signals from this kinase impact numerous downstream processes, positioning AMPK at the core of T-cell metabolic reshaping [88].

Studies have shown that Treg cell differentiation and function are intricately linked to fatty acid oxidation (FAO) [76]. Consequently, it is widely accepted that AMPK promotes the differentiation of Treg cells. In T-cells lacking LKB1, diminished AMPK activity results in heightened glycolytic metabolism and reduced fatty acid oxidation. Likewise, T-cells deficient in LKB1 tend to differentiate into Th1 and Th17 cells in the absence of inflammation [89]. Overall, the influence of the internal environment on the metabolic state and function of CD4+ T-cell subgroups demands further exploration in future studies.

## 5. Conclusions

In summary, current research on atherosclerosis (AS) underscores the distinct biological roles of various T-cell subgroups in AS, particularly the pro-inflammatory effects of Th1/Th17 cells and the anti-inflammatory effects of Th2/Treg cells. Research in immunometabolism shows that T-cell metabolic pathways are essential for their differentiation and function, and targeting these pathways has demonstrated therapeutic potential in treating autoimmune diseases and cancer. However, the connection between T-cell metabolic reprogramming and AS still requires further exploration. Future research will concentrate on the metabolic reprogramming of T-cells in AS, investigating new preventive and therapeutic strategies through modulation of the T-cell microenvironment and metabolic processes. This approach seeks to enhance our understanding of the metabolic foundations of immune function in AS and leverage these insights for clinical advantages.

## Figures and Tables

**Table 1:** Key findings in the references.

Key Findings	References
1. The Role of Pro-inflammatory and Anti-inflammatory T-Cells in Atherosclerosis (AS) Progression	
① Pro-inflammatory Th1 and Th17 cells drive the progression of AS.	[16,17,18,19,20,21,22]
② Anti-inflammatory Th2 and Treg cells can slow down or even reverse AS progression.	[23,24,25,26,27]
2. Metabolic Reprogramming in Pro-inflammatory and Anti-inflammatory T-Cell Differentiation	
Pro-inflammatory T-Cell	
① LDHA and PDK1 are crucial for glucose metabolic reprogramming.	[28,29,30,31,32,33]
② ACC is essential for fatty acid metabolic reprogramming.	[34,35,36,37,38,39,40,41,42,43,44]
③ Amino acid transporters are key in amino acid metabolic reprogramming.	[45,46,47,48,49,50]
Anti-inflammatory T-Cell	
① GLUT 1/3 are critical for glucose metabolic reprogramming.	[51,52,53,54,55,56,57]
② PPAR-γ, FASN, and CPT1 are essential for fatty acid metabolic reprogramming.	[58,59,60,61,62]
③ Arginine and kynurenine are significant in fatty acid metabolic reprogramming.	[63,64]
3. Key Molecular Mechanisms in T-Cell Metabolic Reprogramming	
① mTOR	[65,66,67,68,69,70,71,72,73,74,75,76]
② AMPK	[77,78,79,80,81,82,83,84,85,86,87,88,89]

Abbreviations: LDHA, Lactate Dehydrogenase A; PDK1, Pyruvate Dehydrogenase Kinase 1; ACC, Acetyl-CoA Carboxylase; GLUT 1/3, glucose transporter 1/3; PPAR-γ, peroxisome proliferator-activated receptor γ; FASN, fatty acid synthase; CPT1, Carnitine Palmitoyl transferase I. mTOR, mammalian target of rapamycin; AMPK, AMP-activated protein kinase.

## Data Availability

Not applicable.
